# Septic Embolism in a Patient with Infective Endocarditis and COVID-19

**DOI:** 10.4269/ajtmh.20-1133

**Published:** 2020-10-16

**Authors:** Camila Negreiro Dias, Luís Arthur Brasil Gadelha Farias, Francisco Juliao Moreira Barreto Cavalcante

**Affiliations:** 1 Hospital Universitário Walter Cantídeo (HUWC), Fortaleza, Brazil;; 2 Hospital São José de Doenças Infecciosas (HSJ), Fortaleza, Brazil;; 3 Escola de Saúde Pública do Ceará (ESP/CE), Fortaleza, Brazil

**Figure 1.**
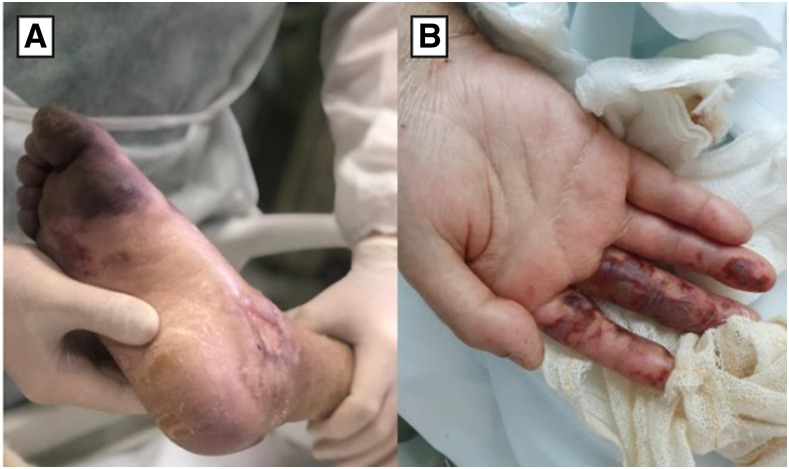
(**A**) Extensive distal infiltrated purpura with necrotic aspect on the right leg. (**B**) Erythematous purpuric macules on the right hand corresponding to a Janeway lesion.

**Figure 2.**
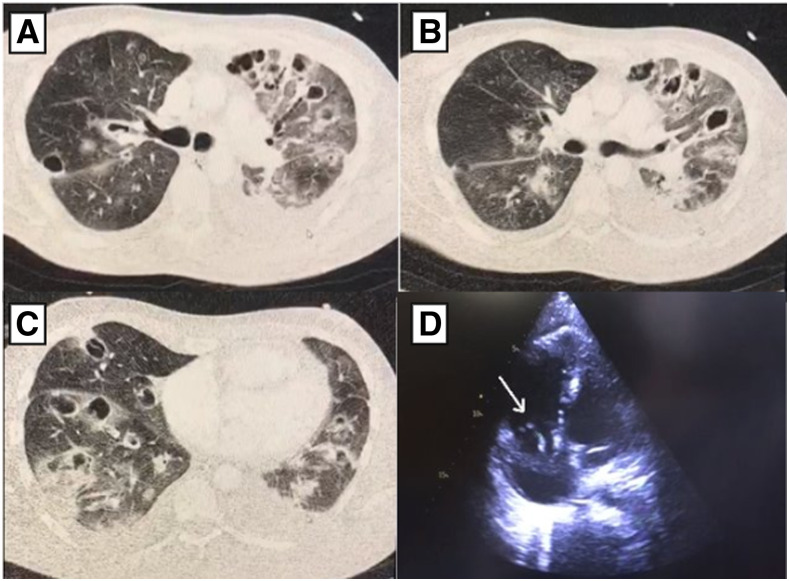
CT pulmonary arteriography. (**A**, **B**, and **C**) Multiple cavitating lesions throughout all lung fields, suggestive of septic pulmonary emboli associated with bilateral glass-ground opacities, and transthoracic echocardiogram. (**D**) Mobile vegetation on the tricuspid valve measuring 5 cm.

A 36-year-old Brazilian patient with a previous history of lower limbs trauma presented to the emergency room with severe respiratory distress and a 7-day history of sudden fever (temperature 37.9°C), cough, and myalgia. On physical examination, his blood oxygen saturation (SPO_2_) level was 89%, with severe respiratory distress, heart rate of 151 beats per minute, and respiratory rate of 29 breaths per minute. On cardiac examination, a pansystolic murmur of grade 3/6 at the left lower sternal area was noted. Skin examination showed an extensive distal infiltrated purpuric macule with necrotic appearance on the right leg, and similar purpuric macules on the right hand, mainly located on the second and third fingers (Figure 1A and B). Because of the severe respiratory distress, the patient required continuous oxygen via nasal cannula 5 L/minute, evolving progressively with clinical worsening, and invasive mechanical ventilation was required. Respiratory secretion was collected from tracheal aspirate and positive for SARS-CoV-2. Computed tomography (CT) pulmonary arteriography demonstrated multiple cavitating lesions throughout all lung fields, suggestive of septic pulmonary emboli associated with bilateral glass-ground opacities (Figure 2A–C). A transthoracic echocardiogram identified a 5 cm mobile vegetation on the tricuspid valve with severe tricuspid regurgitation. Mild pericardial effusion was also present (Figure 2D). During hospitalization, blood culture samples were positive for methicillin-resistant *Staphylococcus aureus.* Laboratory tests showed low hemoglobin (6.9 g/dL), and hematocrit levels (22.9%), leukocytosis (41.990/mm^3^), and normal platelet count range. Ferritin (16.505 mcg/L) was markedly elevated. C-reactive protein (17.4 mg/L) was above reference limits. Troponin, electrolytes, hepatic and renal functions were unremarkable. Embolectomy was not performed because of hemodynamic instability. Antibiotic therapy was started with vancomycin, meropenem, and gentamicin. After 20 days, the patient evolved with clinical worsening and fatal outcome.

Currently, there are few reported cases of infective endocarditis (IE) and COVID-19 infection.^1,2^ Amir et al.^1^ reported the first case of COVID-19 management in a patient with concomitant IE. Other similar cases have been reported, although this association remains poorly understood.^2^ The reported case presented multiple findings of embolic phenomenon. Some studies have suggested that COVID‐19 could also manifest in significant abnormal coagulation parameters, and clinically as vessel thrombosis, with patients being frequently anticoagulated.^3^ Herein, we represent a unique case in which the complication was attributed to IE, although the role of SARS-CoV-2 could not be ruled out. We hypothesized that the area of trauma could be the portal entry of bacteremia. More studies are necessary to understand this association and its management. Full cardiac routine screening should be performed to patients with fever and cardiac abnormalities on physical examination.
